# Joint-Preserving Surgery for Hyperextension Deformity of the Hallux Interphalangeal Joint in a Patient with Rheumatoid Arthritis

**DOI:** 10.1155/2020/5843095

**Published:** 2020-07-08

**Authors:** Takumi Matsumoto, Yuki Shimizu, Song Ho Chang, Taro Kasai, Jun Hirose, Sakae Tanaka

**Affiliations:** Department of Orthopaedic Surgery, Faculty of Medicine, The University of Tokyo, 7-3-1 Hongo, Bunkyo-ku, Tokyo 113-8655, Japan

## Abstract

Interphalangeal hyperextension is one of the major hallux deformities in patients with rheumatoid arthritis; however, there is yet no established surgical method for this deformity. We here present the case of a 69-year-old female patient with rheumatoid arthritis who developed hallux interphalangeal hyperextension and painful callosity on the plantar hallux accompanied by limited dorsiflexion at the metatarsophalangeal joint. Lateral weight-bearing radiograph of the foot revealed misalignment of the medial column and hallux, including a collapsed medial arch, elevated first metatarsal, plantar flexion and deviation of the proximal phalanx, and hyperextension of the distal phalanx. The foot was successfully treated and became symptom-free with opening wedge osteotomy of the medial cuneiform, plantar and proximal translation of the metatarsal head, and tenotomy of the extensor hallucis longus. This case suggests that reconstruction of the sagittal alignment of the medial column and hallux through a combination of osteotomy and soft tissue intervention could be an optional treatment for interphalangeal hyperextension.

## 1. Introduction

The foot is one of the most commonly involved sites early in the course of rheumatoid arthritis (RA) [1]. Foot and ankle involvement increases with the progression of the disease, and >90% of patients with RA reportedly have foot and ankle symptoms at later stages [2, 3]. Although hallux valgus is recognized as the most common feature among rheumatoid foot deformities, other deformities are also observed, including hyperextension of the interphalangeal (IP) joint, which can be symptomatic with a callosity beneath the IP joint [2, 4]. The surgical treatments for hallux valgus in patients with RA have been well described in the literature, with good clinical outcomes reported; however, there has been no consensus about the treatment for hyperextension deformity of the hallux IP joint. In this case report, we introduce our surgical plan and its result in a patient with RA who had the hallux IP hyperextension deformity associated with rocker bottom deformity.

## 2. Case Report

The patient was a 69-year-old female with a 45-year history of seropositive RA. After 42 years of treatment with conventional disease-modifying antirheumatic drugs, her RA disease activity had been well controlled in a state of low disease activity or remission since the introduction of abatacept 500 mg every 4 weeks in combination with methotrexate 4 mg/week and prednisolone 2 mg/day for 3 years. She had previously undergone an arthroplasty in her left radioulnar joint but had no prior surgeries in her foot.

**Figure 1:**
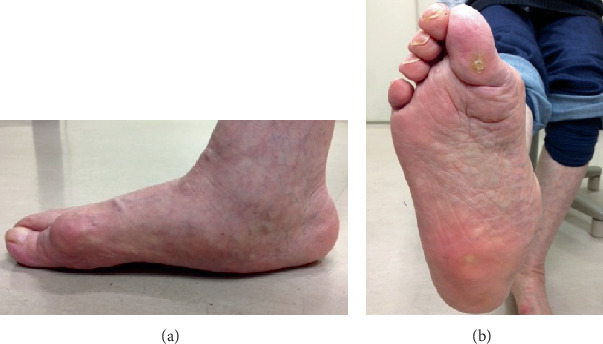
Preoperative photographs of the affected foot. (a) Lateral view of the weight-bearing foot showing rocker bottom deformity, elevated first metatarsal, interphalangeal hyperextension of the hallux, and ground contact at the interphalangeal joint of the hallux. (b) Sole of the foot showing callosity on the plantar surface of the interphalangeal joint of the hallux.

The patient complained of gait disturbance due to a painful callosity in the plantar hallux. The pain of the callosity increased even with conservative treatment with insole use in the past 10 years, and she was referred to our institution for surgical treatment. Her right foot showed severe hindfoot valgus, rocker bottom deformity, and hyperextension of the hallux IP joint in the standing position (Figure 1). The medial forefoot had ground contact not at the first metatarsal head but at the hallux IP joint, which corresponded to the location of the painful callosity. The range of motion at the metatarsophalangeal (MTP) joint was limited to 0° in dorsiflexion and 40° in plantar flexion. She was unable to flex the hallux IP joint actively, suggesting the ruptured or elongated flexor hallucis longus tendon.

**Figure 2:**
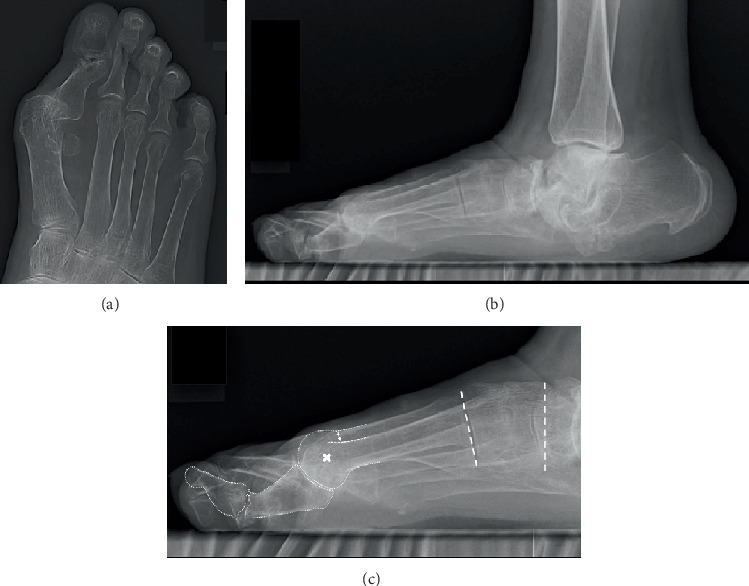
Preoperative weight-bearing radiographs of the foot. (a) Anteroposterior view of the foot showing hallux valgus deformity and no clear space at the metatarsophalangeal joint. (b, c) Lateral view of the foot showing the destruction of the Chopart and subtalar joints, as well as misalignment of the hallux including elevation of the first metatarsal, plantar deviation of the proximal phalanx, and interphalangeal hyperextension. The two-way arrow indicates the distance between two dorsal cortices of the first and second metatarsals, indicating an elevated first metatarsal. The cross indicates the approximate center of the metatarsal head. Dotted lines show the naviculocuneiform and tarsometatarsal joints.

The anteroposterior weight-bearing foot radiograph showed hallux valgus deformity and no clear space at the first MTP joint, but the configuration of the metatarsal head and proximal phalanx was preserved (Figure 2(a)). The findings from the lateral weight-bearing foot radiograph were as follows: (1) severe destruction of the mid- and hindfoot joints and collapse of the medial arch, (2) elevation of the first metatarsal, (3) plantar flexion and deviation of the hallux proximal phalanx, and (4) hyperextension deformity of the hallux distal phalanx (Figures 2(b) and 2(c)).

The treatment options were discussed with the patient. She rejected our recommendation to perform triple arthrodesis for reconstructing the mid- and hindfoot deformities because of the need for prolonged non-weight-bearing during the postoperative course. She strongly hoped to undergo a surgery restricted to the forefoot, which would allow her to ambulate with the heel immediately postoperatively. After an adequate consultation with the patient and her family, including about the possibility of recurrent toe deformities due to the possible progression of mid- and hindfoot deformities, we decided to perform surgery that involved correcting the sagittal alignment of the hallux in order to relieve the plantar pressure at the IP joint.

### 2.1. Surgery and Postoperative Course

**Figure 3:**
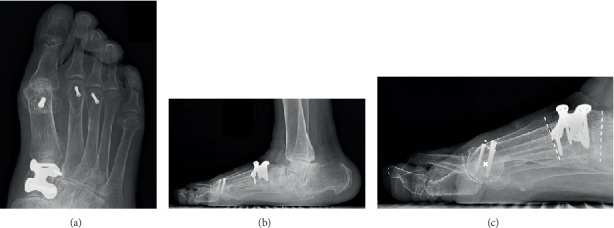
Postoperative weight-bearing radiographs of the foot. (a) Anteroposterior view of the foot showing improved hallux valgus deformity and congruent metatarsophalangeal joint. (b, c) Lateral view of the foot showing improvement of the elevated first metatarsal, plantar deviation of the proximal phalanx, and interphalangeal hyperextension. The two arrowheads indicate the distance between two dorsal cortices of the first and second metatarsals, indicating improvement of the elevated first metatarsal compared with Figure 2(c). The cross indicates the approximate center of the metatarsal head. Dotted lines drawn along the naviculocuneiform and tarsometatarsal joints demonstrate the improvement of plantar flexion by approximately 10° after the Cotton osteotomy.

**Figure 4:**
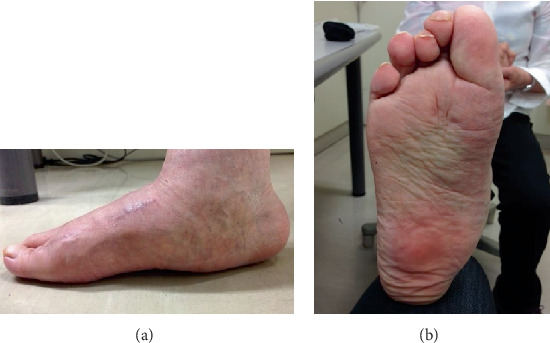
Postoperative photographs of the foot 44 months after the primary surgery. (a) Lateral view of the weight-bearing foot showing the ground contact at the first metatarsal head. (b) Sole of the foot showing the disappearance of the callosity on the plantar surface of the first interphalangeal joint.

The patient was placed in the supine position, and a thigh tourniquet was applied. A 2 cm dorsal longitudinal incision was made over the medial cuneiform between the extensor hallucis longus and extensor hallucis brevis tendons, and a linear capsulotomy was made to expose the underlying medial cuneiform. About 10° of opening wedge osteotomy was applied to the medial cuneiform using a lamina spreader as an opener to an extent that the spreader did not crush the bone, and about 10° of opening was achieved. The osteotomy site was fixed using a dorsal locking plate after filling the gap with granulated *β*-tricalcium phosphate. Thereafter, a dorsal longitudinal incision was made at the first MTP joint. A capsular incision was then made in a longitudinal orientation a few millimeters laterally away along the medial edge of the extensor hallucis longus tendon. After the dorsal, medial, and lateral capsules and collateral ligaments were released, the adhesions of the plantar plate were released using the McGlamry elevator. A distal oblique osteotomy was performed at the metatarsal head in a direction from the dorsal-distal to the plantar-proximal aspect with an angle of 45° to the metatarsal shaft. The metatarsal head was pushed backward by 1 cm and laterally by 5 mm. After a provisional fixation of the osteotomy site with a Kirschner wire, the lid of the instrument set was placed on the sole of the foot to simulate weight-bearing. It was confirmed that the area of the sole underneath the first metatarsal head touched the lid and the dorsiflexion at the MTP joint increased up to 50°. The metatarsal head was fixed with a headless compression screw, and the dorsal prominent bone of the proximal fragment was excised. As the tension of the extensor hallucis longus was tight, the tendon was cut at the attachment site on the distal phalanx, folded back, and sutured to itself after penetrating through the distal part of the dorsal capsule. Additionally, Weil osteotomies for the second and third metatarsals and percutaneous flexor digitorum longus tenotomy at the plantar distal IP joint of the fourth and fifth toes were performed for coexisting lesser toe deformities. The patient was allowed to bear weight on the heel on the next day after the surgery by using a heel weight-bearing shoe, and foot-flat gait was permitted at 3 weeks postoperatively. Normal ambulation, including the toe push-off phase, in a normal shoe, was allowed at 10 weeks after the surgery when bone union at all osteotomy sites was confirmed. At the latest follow-up at 44 months postoperatively, the patient had no complaints of pain and was satisfied with the result. No progression of hindfoot destruction and flatfoot deformity has occurred (Figure 3). IP joint dorsiflexion angle, the angle between the longitudinal axis of the first distal phalanx and that of the first proximal phalanx, was slightly improved from 43 degrees preoperatively to 36 degrees at the latest follow-up. The operated foot showed the disappearance of the callosity on the plantar hallux IP joint and ground contact at the first metatarsal head in the standing position (Figure 4). The range of motion was up to 40° in dorsiflexion and 30° in plantar flexion at the MTP joint.

## 3. Discussion

The three major hallux deformities in patients with RA are hallux valgus, hallux rigidus, and IP hyperextension, according to the observation of 200 consecutive patients with RA by Kirkup et al. [4]. Among these deformities, hallux rigidus and IP hyperextension are closely related [4, 5]. Kirkup et al. reported that half of the cases with hallux rigidus were accompanied by IP hyperextension and considered IP hyperextension as a compensatory change for the limited dorsiflexion caused by hallux rigidus [4]. Another study classifying the hallux deformities of 527 RA feet into five clusters demonstrated that the boutonniere deformity, characterized by an elevated first metatarsal, plantar displacement of the proximal phalanx, and IP hyperextension, was correlated with a decreased longitudinal arch [5]. The study also demonstrated that the plantar displacement of the proximal phalanx was significantly correlated with decreased dorsiflexion in the MTP joint and speculated that the boutonniere deformity was a result of compensatory hyperextension at the IP joint for the limited dorsiflexion at the MTP joint [5], which can be explained by the reverse windlass mechanism in the flatfoot proposed by Hicks [6].

There is yet no established surgical treatment for boutonniere deformity of the hallux in the RA foot. Only a few studies have been published on this topic, which reported on simultaneous arthrodesis of the MTP and IP joints [7, 8]. A study of six cases of simultaneous arthrodesis of the MTP and IP joints involving five patients with RA reported no limitation in daily activities with painless hallux but described limitations interfering with full athletic activities and concluded that this method is a reasonable option for patients with moderate demands [8]. Conversely, arthrodesis for progressive IP joint arthritis after MTP joint arthrodesis has been reported to have many problems [9]. A study comparing patients who underwent IP joint arthrodesis with and without prior ipsilateral first MTP joint arthrodesis demonstrated higher nonunion and complication rates in patients with prior first MTP joint arthrodesis than in those without (35.3% vs. 8.0% and 52.9% vs. 24.0%, respectively) [9]. Given the importance of IP joint function in helping the foot compensate for lost MTP joint function, arthrodesis of the IP joint in a situation of limited MTP joint motion would be troublesome even after a successful bone union, as it would interfere with the toe-off phase of gait or with shoe wearing. IP joint arthrodesis with a mobile MTP joint, such as interpositional arthroplasty or silicone implant arthroplasty, is a possible alternative; however, it could lead to cock-up deformity, push-off weakness, infection, silicone synovitis, and osteolysis [10, 11]. Moreover, considering that the configuration of the MTP joint was preserved in the present case, we chose to gain motion at the MTP joint by restoring the positional abnormal relation between the plantarily deviated proximal phalanx and the elevated metatarsal head. Reconstruction of the collapsed mid- and hindfoot deformities was supposed to be prioritized over reconstruction of the hallux; however, this carries the risk of postoperative immobilization or restricted weight-bearing for a prolonged period, which might be undesirable in elderly patients or patients with RA who already have limited ability to perform daily life activities. The extension of the IP joint was only partially recovered probably because of the ruptured or elongated flexor hallucis longus tendon. The success of the present case indicated that the key of the surgical treatment to relieve the overload at the IP joint was to release the limited range of motion at the MTP joint but not the deformity correction of the IP joint.

Plantar flexion opening wedge medial cuneiform osteotomy, known as the Cotton osteotomy, is an effective procedure to lower an elevated first metatarsal and has an advantage of adjusting the degree of plantar flexion according to the size of the opened wedge [12]. Although there is still no information about the limitation of correctable angle with this procedure, the case series involving 15 patients (16 feet) who underwent the Cotton osteotomy procedure reported an average improvement of 9° in the lateral first metatarsal-medial cuneiform angle, which was considered to reflect the corrective angle achieved at the osteotomy site [13]. The Cotton osteotomy makes the medial column longer through the nature of opening wedge osteotomy, leading to increased tension at the MTP joint, which is disadvantageous for the aim of gaining range of motion. In the present case, we needed to add a distal oblique shortening osteotomy of the metatarsal head in order to release the tension at the MTP joint and compensate for the shortage of plantar translation of the metatarsal head after the Cotton osteotomy. Distal oblique osteotomy of the metatarsal head has an advantage of being able to adjust the priority between shortening and plantar translation of the metatarsal head by changing the osteotomy angle, although care should be taken not to make the exit of the plantar cut distal to the capsular attachment in order to protect the plantar blood supply [14]. Proximal plantar closing wedge osteotomy of the first metatarsal might be one of the alternatives to achieve both shortening and plantar translation of the metatarsal head, although the adjustment of each priority might be difficult [15].

## 4. Conclusions

We described a case of RA suffering from hyperextension deformity of the hallux interphalangeal joint, which was successfully treated and became symptom-free with opening wedge osteotomy of the medial cuneiform, plantar and proximal translation of the metatarsal head, and tenotomy of the extensor hallucis longus. The procedure described in the present report represents a potential option in surgical planning for IP hyperextension of the hallux in patients with RA.

## Data Availability

The medical data used to support the findings of this study have not been made available because they belong to the patient and are not permitted to use except for this case report.
